# Occurrence and Antimicrobial Susceptibility Profiles of *Streptococcus equi* subsp. *zooepidemicus* Strains Isolated from Mares with Fertility Problems

**DOI:** 10.3390/antibiotics11010025

**Published:** 2021-12-27

**Authors:** Francesca Paola Nocera, Elena D’Eletto, Monica Ambrosio, Filomena Fiorito, Ugo Pagnini, Luisa De Martino

**Affiliations:** 1 Department of Veterinary Medicine and Animal Production, University of Naples “Federico II”, Via F. Delpino 1, 80137 Naples, Italy; elena.deletto93@gmail.com (E.D.); monica.ambrosio@unina.it (M.A.); filomena.fiorito@unina.it (F.F.); upagnini@unina.it (U.P.); ldemarti@unina.it (L.D.M.); 2 Task Force on Microbiome Studies, University of Naples “Federico II”, Naples, 80137, Italy

**Keywords:** *Streptococcus equi* subsp. *zooepidemicus*, antibiotic resistance, mares, endometritis

## Abstract

*Streptococcus equi* subsp. *zooepidemicus* (*S. zooepidemicus*), is a β-hemolytic *Streptococcus* belonging to the Lancefield group C; it is a rare human pathogen, but in horses, it is frequently associated with endometritis. This study aimed to isolate *S. zooepidemicus* strains, associated with bacterial endometritis in mares, and to define their antimicrobial resistance profile. Twenty-three isolates were recovered from one hundred ninety-six equine uterine swabs (11.7%). Bacterial identification was carried out by Api 20 Strep and confirmed by matrix assisted laser desorption ionization time of flight mass spectrometry (MALDI-TOF-MS), while antimicrobial susceptibility testing was performed by disk diffusion method on Muller Hinton agar plates. The antibiotic resistance profiles of the isolates revealed a high percentage of resistance to amikacin (95.6%), ampicillin (73.9%) and tetracycline (69.6%), while ceftiofur and ceftriaxone were highly effective with 82.6% and 78.3% of the isolates inhibited, respectively. An intriguing value of resistance to penicillin (34.8%), which represents the first-choice antibiotic in equine *S. zooepidemicus* infections, was observed. Furthermore, a high prevalence of multidrug-resistant strains (82.6%) was recorded. Continuous surveillance of this potential zoonotic pathogen and an appropriate antimicrobial stewardship program with the promotion of correct use of antimicrobials, after a proper diagnosis, are needed to allow an effective therapy.

## 1. Introduction

*Streptococcus equi* subsp. *zooepidemicus* (*S. zooepidemicus*), is a β-hemolytic streptococcus belonging to the Lancefield group C. It is a rare human pathogen, but it is commonly isolated from bacterial infections in a wide range of animal species like horses [1], dogs [2,3], felines [4,5,6], swine [7,8], guinea pigs [9], ruminants [10,11], camelids [12] and non-human primates [13].

In mares, *S. zooepidemicus* is one of the main causative agents of infectious endometritis [1,14]. Moreover, being an opportunistic pathogen, it can be isolated in horses suffering from respiratory infections, which generally occur in presence of stressful conditions, such as transport, high temperatures, or concomitant viral infections [15].

Furthermore, *S. zooepidemicus* zoonotic potential should not be underestimated since, even if in rare cases, it can be responsible for serious diseases in humans, such as sepsis, septic arthritis, meningitis and pneumonia [16,17]. According to literature, human *S. zooepidemicus* infections are usually due to direct contact with infected horses [16,17,18] or infected dogs [19,20], but there are also reports about the consumption of contaminated food of animal origin, such as cheese or milk [21,22].

Referring to infectious endometritis in mares, *S. zooepidemicus* ability to cause endometritis depends on different factors, which can be associated both with uterine defense mechanisms and with the pathogenic potential of the involved causative agent. Indeed, Hughes and Loy [23] experimentally infected mares with *S. zooepidemicus*, demonstrating that mares can be classified as susceptible or resistant to infectious endometritis based on their ability to resist infection. In mares, susceptible to bacterial uterine infection, uterine clearance is generally impaired, as a consequence of anatomical dysfunctions, such as a pendulous uterus, tight cervix, or impaired myometrial contractility, thus resulting in susceptibility to aspirating air or accumulating fluid or urine in the vagina and uterus [24,25,26], whilst mares with a competent immune response and functional anatomy of the reproductive tract are able to clear infections spontaneously [26]. It is worth noting that infectious endometritis is not caused solely by random contamination of the caudal reproductive tract by *S. zooepidemicus*, but it is likely to be caused by more specialized endometrial pathogenic strains. Rasmussen et al. [27] showed that *S. zooepidemicus* strains isolated from the uterus of mares suffering from infectious endometritis shared a high genetic relatedness compared to isolates obtained from the caudal reproductive tract (vagina and clitoral fossa), highlighting the presence of a genetic subpopulation of *S. zooepidemicus* strains associated with infectious endometritis. Therefore, it appears clear that some strains adapt better than others to survive and colonize the endometrial environment.

*S. zooepidemicus* is usually responsible for a dormant subclinical infection, persisting in endometrium tissue and resisting the immune system and antimicrobial treatment. This is related to *S. zooepidemicus* ability to survive in epithelial cells in the form of “persister” cells [28]. “Persister” bacterial cells are a subpopulation of cells with a dormant phenotype, tolerant to antibiotic treatment and other environmental stressors and that are able to resume normal growth in a stochastic manner [29].

Besides *S. zooepidemicus* “persister” cells’ ability to resist antibiotics, in veterinary medicine the circulation of multidrug-resistant streptococci including *S. zooepidemicus* has increased over the years, due to the abuse and misuse of available antibiotics. Generally, β-lactams and aminoglycosides are commonly prescribed to treat streptococcal infections, since they are broad-spectrum antibiotics. Particularly, penicillin has always been considered the first-choice antibiotic in equine *S. zooepidemicus* infections, due to its good efficacy and low levels of resistance; whilst aminoglycosides have reduced efficacy against streptococci when administered in low concentrations, which is due to their restricted drug uptake [30]. However, it has been reported that the combination of aminoglycosides with other antibiotics, such as penicillins and glycopeptides turns out to have a significant bactericidal synergy [30]. In addition, low levels of resistance to aminoglycosides are generally detected in most *Streptococcus* spp., and high levels of resistance to these antibiotics are observed not very frequently [31]. Fluoroquinolones and cephalosporins are also used for the treatment of streptococcal infections, but they should be administered based on antimicrobial susceptibility testing, due to their critical importance as antibiotics [31]. Differently, *S. zooepidemicus* and in general streptococci show often high levels of resistance to tetracyclines [31]. Thus, in this multidrug resistance scenario, it is important to warrant continued monitoring of *S. zooepidemicus* susceptibility profiles to antibiotics commonly used in clinical veterinary practice. Indeed, the increased spread of multidrug-resistant isolates to a number of commonly used antibiotics reinforces the need for an appropriate antimicrobial stewardship program with the promotion of correct and responsible use of antimicrobials, to allow effective therapy.

The purpose of this study was to define the prevalence of *S. zooepidemicus* strains-associated infectious endometritis and their antimicrobial susceptibility profiles isolated in mares suffering from fertility problems in the year 2018.

## 2. Results

### 2.1. S. zooepidemicus Colonies Identification

From a total of one hundred ninety-six uterine swabs, twenty-three isolates of *S. zooepidemicus* were detected with a prevalence of 11.7%. Only four (17.4%) samples gave bacterial growth on solid media without requiring enrichment, while the other isolates (19 strains, 82.6%) were obtained only after the broth enrichment step. This is probably a consequence of a low bacterial load in transport medium samples.

The suspected twenty-three *S. zooepidemicus* isolates were all detected in pure cultures and grew by forming β-hemolytic colonies on CNA agar plates. Furthermore, all the recovered isolates resulted to be catalase and oxidase negative, and they appeared as Gram-positive cocci organized in chains on the smears.

### 2.2. S. zooepidemicus Isolates Identification by Api 20 Strep and MALDI-TOF-MS

Api 20 Strep identification results were *Streptococcus equi* subsp. *zooepidemicus* (probability, 99.9%) for all recovered isolates. The phenotypic bacterial identification was further confirmed by proteomic profiling performed by matrix-assisted laser desorption ionization time of flight mass spectrometry (MALDI-TOF-MS) (Bruker Daltonics, Bremen, Germany). Precisely, all *S. zooepidemicus* isolates were identified at subspecies level by MALDI- TOF-MS with a log(score) range 2.2–2.6. Table 1 summarizes the obtained identification results by Api 20 Strep and MALDI-TOF-MS.

### 2.3. Phenotypic Antibiotic Resistance Profiles of S. zooepidemicus Isolates

**Figure 1:**
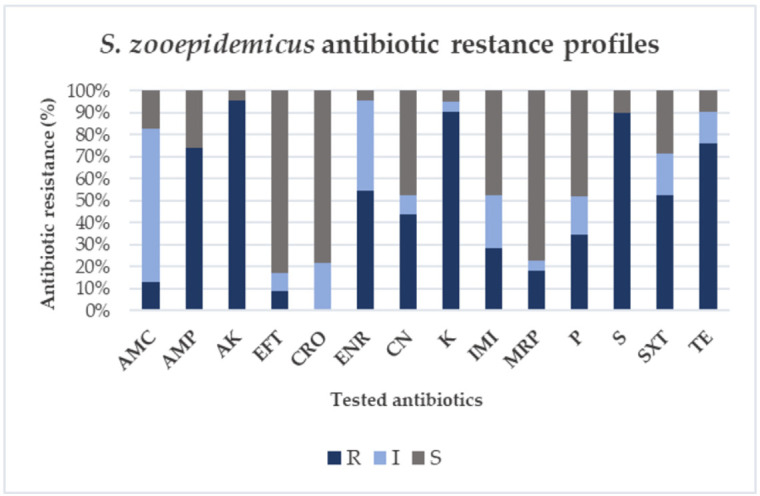
Antibiotic resistance profiles of 23 *S. zooepidemicus* isolates. The isolates were classified as resistant (R), intermediate (I), or susceptible (S) to tested antibiotics. Antibiotics: AMC: amoxicillin-clavulanate; AMP: ampicillin; AK: amikacin; EFT: ceftiofur; CRO: ceftriaxone; ENR: enrofloxacin; CN: gentamicin; K: kanamycin; IMI: imipenem; MRP: meropenem; P: penicillin; S: streptomycin; SXT: sulfamethoxazole-trimethoprim; TE: tetracycline. Since 22 strains were tested to ENR, 21 to K, 22 to IMI, 22 to MRP, 20 to S, 21 to SXT and 21 to TE, a maximum error of 4.3% (1/23 × 100 = 4.3%) was considered.

The antimicrobial susceptibility results of the twenty-three isolates, obtained by Kirby-Bauer disk diffusion testing, are reported in Figure 1 and in Supplementary Materials. The highest levels of resistance were recorded for amikacin with a value of 95.6% (22/23 strains) followed by kanamycin with a value for both antibiotics of 82.6% (19/23 strains), streptomycin (78.2%; 18/23 strains), ampicillin (73.9%; 17/23 strains), tetracycline (69.5%; 16/23 strains) and enrofloxacin (52.1%; 12/23 strains), whilst only third generation cephalosporins, such as ceftiofur and ceftriaxone, were highly effective with 82.6% and 78.3% of the isolates inhibited, respectively. Low resistance values were recorded also for sulfamethoxazole-trimethoprim (47.8%; 11/23 strains), gentamicin (43.4%; 10/23 strains). High values of intermediate susceptibility were recorded for amoxicillin—clavulanate (69.5%; 16/23 strains). Since not all samples were tested to all antibiotics, for the estimation of the percentage of resistance, susceptibility, or intermediate susceptibility to antibiotics, such as enrofloxacin, kanamycin, imipenem, meropenem, streptomycin, sulfamethoxazole-trimethoprim and tetracycline, a maximum error of 4.3% (1/23 × 100 = 4.3%) was considered. 

An alarming result was represented by the high prevalence of multidrug-resistant strains with 82.6% of the total isolates, showing resistance to more than three classes of antimicrobials.

**Figure 2:**
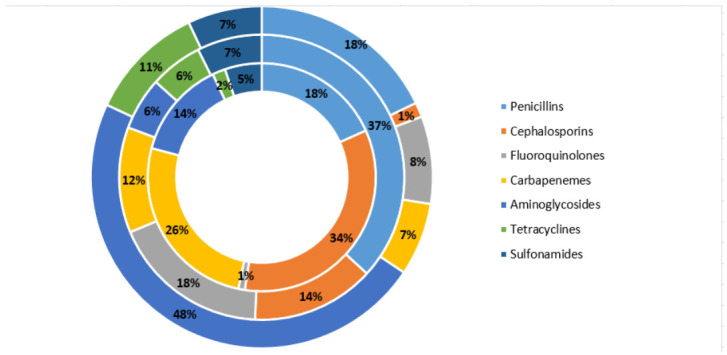
Distribution of the percentage of resistance, intermediate susceptibility, susceptibility for the tested antibiotic classes. Resistance is reported in the outermost circle, intermediate susceptibility is reported in the middle circle, susceptibility is reported in the inner circle.

Referring to the antibiotic classes as reported in Figure 2, higher levels of resistance were observed for aminoglycosides (48%), penicillins (18%), and tetracycline (11%), while lower levels were found for fluoroquinolones (8%), carbapenems (7%), sulfonamides (7%) and cephalosporins (1%). The class with the greatest susceptibility is represented by cephalosporins (34%) followed by carbapenems (26%), penicillins (18%), aminoglycosides (14%), while lower percentages were found for sulfonamides (5%), tetracycline (2%) and fluoroquinolones (1%). The class with the highest frequency of intermediate was the one of penicillins (37%), followed by fluoroquinolones (18%), cephalosporins (14%), carbapenems (12%), sulfonamides (7%), tetracycline (6%) and aminoglycosides (6%).

## 3. Discussion

In the present study the frequency of isolation of *S. zooepidemicus* from uterine swabs, obtained by mares suffering from endometritis, was evaluated. Moreover, the association between infection and antibiotic resistance profiles of the isolated strains was investigated. Indeed, from one hundred ninety-six swabs examined in 2018, twenty-three resulted to be positive for *S. zooepidemicus*, with a prevalence of 11.7%. Precisely, only four (17.4%) samples gave bacterial growth on solid media without requiring enrichment, while the other isolates (19 strains, 82.6%) were obtained only after the broth enrichment step. *S. zooepidemicus* is able to deeply infect the endometrium [27,32], so by using a uterine swab, it is possible not to succeed in detecting the presence of this pathogen, as uterine swabs come into contact only with a limited part of the luminal endometrial surface [33]. Consequently, the prevalence of isolation of *S. zooepidemicus* could be underestimated, if the additional phase of broth enrichment culture is not performed before sowing samples on CNA agar plates [14]. In particular, the obtained results, with nineteen samples requiring enrichment before plating on CNA solid medium, suggest a condition of subclinical endometritis and this may be also indicative of a low bacterial load in the starting sample. Furthermore, this result can be justified by *S. zooepidemicus’* ability to survive in endometrium by forming “persistent” or dormant cells [28], explaining in part why some *S. zooepidemicus* strains can cause persistent subclinical infections in mares [34], leading to fertility problems.

*S. zooepidemicus* and *Streptococcus equi* subp. *equi* are frequently isolated from horses and being closely related, they can be misidentified. For this reason, in this study, the biochemical Api 20 Strep identification of the recovered isolates was confirmed by MALDI-TOF-MS analysis, which always gave (log) score ≥ 2.2 at subspecies level identification. Thus, MALDI-TOF-MS can be considered a useful, reliable, and rapid technique for *S. zooepidemicus* identification, able to discriminate it from *Streptococcus equi* subsp. *equi*, the causative agent of strangles in horses, as already reported in the literature [35,36].

In this study, 82.6% of the total *S. zooepidemicus* isolates displayed multidrug-resistance profiles, showing resistance to more than three antibiotic classes. Our result agrees with many other studies, which report an increasing trend of multidrug resistance associated with *S. zooepidemicus* strains over the years [37,38]. High levels of resistance were recorded for amikacin (95.65%), followed by ampicillin (73.91%). Moreover, a considerable number of strains were found to be resistant to tetracycline (69.56%) as expected, enrofloxacin (52.17%) and gentamicin (43.48%). Particularly, the highest levels of resistance were observed for amikacin and this result appears to be in line with the data reported by Pisello et al. [38], with only 2.1% of the isolates susceptible to this antibiotic. However, the high levels of resistance (48%) recorded for aminoglycosides are not surprising, since they are known to be poorly effective against aerobic Gram-positive bacteria [39] and this may explain the high value of resistance observed. Differently from aminoglycosides, *S. zooepidemicus* isolates exhibited variable susceptibility values for β-lactam antibiotics. A high value of resistance was recorded for ampicillin (73.91%). Penicillin, commonly used for the treatment of β-hemolytic streptococci infections [40], showed an intriguing percentage of resistance of 34.8%. This recorded result agrees with the one reported by another Italian study [38], that underlined a decrease of penicillin efficacy against *S. zooepidemicus* isolates. However, it is worth noting that penicillin still displays a good efficacy against *S. zooepidemicus* isolates, even though the antibiotic susceptibility results may be influenced by the selective pressure of different geographical locations. Amoxicillin-clavulanate had the lowest levels of resistance (13.04%) but it was the antibiotic with the highest intermediate susceptibility value (69.56%), making, in this study, penicillins the first antibiotic class with the highest frequency of intermediate isolates (37%).

Referring to cephalosporins, the antibiotic class with the highest susceptibility rate (34%)*, S. zooepidemicus* isolates showed high susceptibility to ceftiofur (82.6%) and ceftriaxone (78.26%). In horses, ceftiofur is generally used to treat lower respiratory tract infections caused by *S. zooepidemicus* [41,42]. Due to its high efficacy and being considered as a third-generation cephalosporin of critical importance in human medicine [43], it should be administered prudently based on antimicrobial susceptibility testing.

Regarding fluoroquinolones, the second antibiotic class with the highest intermediate susceptibility rate (18%), enrofloxacin presented low levels of susceptibility (4.35%). Fluoroquinolones, as well as third-generation cephalosporins, are considered by World Health Organization (WHO) as critically important antimicrobials in human medicine, classified with the priority criterion 1 [43]. Consequently, the growing percentage of resistance against these antibiotics represent a serious concern of public health with important implication for clinical practice both in human and veterinary medicine [44,45]. Traditionally sulfonamides are considered to have good antimicrobial activity against *S. zooepidemicus*, with low resistance rates as stated by Feary et al. [46]. Contrary to the current study, the percentages of resistant strains are higher than those of susceptible strains, with values of 47.8% and 26%, respectively. To face the alarming growing circulation of multidrug-resistant strains, equine endometritis empirical therapy should be avoided, preferring treatments based on antimicrobial susceptibility testing results.

In addition, *S. zooepidemicus* is not only a relevant veterinary pathogen but also of zoonotic importance, with horses acting as main reservoirs for humans, particularly horse personnel and veterinarians [47,48]. Thus, *S. zooepidemicus*-associated infections could be considered as zoonoses [49,50], since its transmission has been documented also by the consumption of unpasteurized milk and dairy products [21,22], but also from other ill animals, such as domestic dogs [19,20] and guinea pigs [9]. Thus, further studies on risk factors on its zoonotic transmission are needed. Although *S. zooepidemicus* is a rare zoonotic pathogen in humans, older people may have a higher risk for serious or fatal outcomes from infection; in 32 reported cases, the median age was 61 with seven deaths (case-fatality rate = 22%) [16]. Therefore, it is strongly recommended to wash hands with soap and water after contact with horses or other animals. Moreover, further studies on risk factors on its zoonotic transmission and on the spectrum of human diseases associated with *S. zooepidemicus* are needed.

## 4. Materials and Methods

### 4.1. Sampling

In 2018, a total of one hundred ninety-six uterine swabs were collected by veterinarians specialized in equine reproduction from mares diagnosed with suspected bacterial endometritis. Precisely, mares enrolled in this study were 4 to 21 years old (mean age 12.5 years) and belonged to different breeds (Italian Trotter: 85; Quarter Horse: 50; English Thoroughbred: 30; Arabian: 26; Holstein: 5). Clinical inclusion criteria were represented by a history of repetitive infertility, resorption/abortion, repeated breeding after artificial insemination, fluid in uterine lumen during the luteal phase and/or vulval discharge.

For bacteriological examination, before collecting the uterine swabs, the external genitals of mares were rinsed twice with a 0.1% solution of iodopovidone and dried. After that, a sterile swab with double protection was introduced in the uterus through the cervix of estrous mares by a gloved hand, and it was advanced beyond the protective sheath until it contacted the endometrium, where it was softly rotated. Then, the inner swab was withdrawn into the outer sheath, and the entire culture instrument was retired. Once outside the mare, the swab tip was placed in a Stuart agar gel transport medium (Aptaca Spa, Asti, Italy). All the collected uterine swabs were labeled and transported within 24 h, by using an icebox, to the Microbiological Diagnostic Laboratory of the Department of Veterinary Medicine and Animal Production of the University of Naples “Federico II “, where they were processed. Since all the performed veterinary investigations were clinically needed, the Ethical Committee approval was not required.

### 4.2. S. zooepidemicus Isolation

Equine uterine swabs were cultured and plated in parallel on different solid agar media and in broth-enrichment Brain Heart Infusion (BHI) and incubated aerobically at 37 °C for 24 h. The day after, turbid BHI tubes were sub-cultured on the same agar plates. Precisely, *S. zooepidemicus* isolation was performed by using Columbia blood agar (CNA) with 5% sheep blood, a selective medium for Gram-positive bacteria isolation. Once bacterial growth was detected on CNA agar plates, the isolated strains were first screened by using standard, rapid techniques: colony morphology, cellular morphology by Gram’s staining method, catalase and oxidase tests.

### 4.3. S. zooepidemicus Isolates Identification by Api 20 Strep and MALDI-TOF -MS

For an initial identification, a biochemically-based commercial system, manual Api 20 Strep (bioMériuex, Marcy L’Etoile, France) was performed. For the interpretation of results, the manufacturer’s guidelines were followed. Precisely, probability values of >90%, >80% indicated excellent and good levels of identification, respectively. Probability values < 60% indicates a not reliable identification.

The recovered isolates were then identified also by MALDI-TOF MS analysis (Bruker, Daltonics, Germany), to confirm Api 20 Strep results. For MALDI-TOF-MS identification, fresh colonies were used. The protocol used was the following: the bacterial colony was first inoculated in a MALDI-TOF-MS target plate. Subsequently, 1 µL of cinnamic acid matrix solution was added to the sample and dried at room temperature for ten minutes. Afterward, the target plate was placed in the equipment for MALDI-TOF-MS analysis. The identification was based on the score values released by the equipment’s instructions. Specifically, according to Bruker biotyper’s guidelines, a score of ≥2.0 indicated a highly probable species-level identification, a score of 1.70 to 1.99 indicated a secure identification to the genus level, and a score of <1.7 was interpreted as no identification.

*S. zooepidemicus* ATCC^®^ 53698^TM^ was included as positive control strain.

### 4.4. Antibiotic Susceptibility Testing

Antibiotic resistance profiles of the isolated strains were evaluated by the disk diffusion method on Mueller Hinton agar plates with 5% sheep blood (Liofilchem, Teramo, Italy). All isolates were tested for their susceptibility to 14 antibiotics, belonging to seven different classes: amoxicillin—clavulanate (AMC, 20/10 µg), amikacin (AK, 30 µg), ampicillin (AMP,10 µg), ceftiofur (EFT, 30 µg), ceftriaxone (CRO, 30 µg), enrofloxacin (ENR, 5µg), gentamicin (CN, 10 µg), kanamycin (K, 30 µg), imipenem (IMI, 10 µg), meropenem (MRP, 10 µg), penicillin (P, 10 IU), streptomycin (S, 10 µg), sulfamethoxazole-trimethoprim (SXT, 1.25/23.75 μg), tetracycline (TE, 30 µg). The isolates were classified as susceptible (S), intermediate (I), or resistant (R) according to the Clinical and Laboratory Standards Institute [51] and to the European Committee on Antimicrobial Susceptibility Testing [52] guidelines for *Streptococcus* spp. Furthermore, antibiotic choice was performed also considering the antibiotics most commonly used in equine clinical practice, based on their efficacy against uterine infections.

*S. zooepidemicus* isolates, showing resistance to at least three different antibiotic classes, were defined multidrug-resistant strains according to [53].

The tested antibiotics and the classes they belong to are reported in Table 2.

### 4.5. Data Management and Descriptive Statistical Analysis

All diagnostic results generated by the Microbiological Diagnostic Laboratory were recorded and entered into a Microsoft 365 Excel™ spreadsheet for successive analysis.

Descriptive statistical analysis was employed to analyze the prevalence of *S. zooepidemicus* and the frequencies of antibiotic resistance among recovered isolates. Referring to antibiotic resistance frequencies, since not all isolates were tested to all antibiotics, for the estimation of the percentage of resistance, susceptibility, or intermediate susceptibility a maximum error of 4.3% (1/23 × 100 = 4.3%) was considered. All graphics were made with Excel software.

## 5. Conclusions

The increasing spread of multidrug-resistant *S. zooepidemicus* has become a relevant veterinary issue, highlighting the need for continuous surveillance of this pathogen. Moreover, a proper diagnosis and an effective therapeutic approach are needed to allow a rapid and effective antimicrobial treatment. In this scenario, the implementation of a stewardship program should be supported, to ensure responsible and prudent use of antimicrobials in equine veterinary practice.

In addition, the zoonotic potential of *S. zooepidemicus* should be further investigated, in order to provide guidelines on best practices to limit the spread of disease within horse facilities and to the public.

## Figures and Tables

**Table 1:** Api 20 Strep and MALDI-TOF-MS *S. zooepidemicus* identification results.

Isolate	Api 20 Strep Identification *	MALDI-TOF- MS Identification **
1	*Streptococcus equi* subsp. *zooepidemicus* (99.9%)	*Streptococcus equi* subsp. *zooepidemicus* (2.3)
2	*Streptococcus equi* subsp. *zooepidemicus* (99.9%)	*Streptococcus equi* subsp. *zooepidemicus* (2.2)
3	*Streptococcus equi* subsp. *zooepidemicus* (99.9%)	*Streptococcus equi* subsp. *zooepidemicus* (2.4)
4	*Streptococcus equi* subsp. *zooepidemicus* (99.9%)	*Streptococcus equi* subsp. *zooepidemicus* (2.5)
5	*Streptococcus equi* subsp. *zooepidemicus* (99.9%)	*Streptococcus equi* subsp. *zooepidemicus* (2.5)
6	*Streptococcus equi* subsp. *zooepidemicus* (99.9%)	*Streptococcus equi* subsp. *zooepidemicus* (2.4)
7	*Streptococcus equi* subsp. *zooepidemicus* (99.9%)	*Streptococcus equi* subsp. *zooepidemicus* (2.5)
8	*Streptococcus equi* subsp. *zooepidemicus* (99.9%)	*Streptococcus equi* subsp. *zooepidemicus* (2.6)
9	*Streptococcus equi* subsp. *zooepidemicus* (99.9%)	*Streptococcus equi* subsp. *zooepidemicus* (2.4)
10	*Streptococcus equi* subsp. *zooepidemicus* (99.9%)	*Streptococcus equi* subsp. *zooepidemicus* (2.5)
11	*Streptococcus equi* subsp. *zooepidemicus* (99.9%)	*Streptococcus equi* subsp. *zooepidemicus* (2.6)
12	*Streptococcus equi* subsp. *zooepidemicus* (99.9%)	*Streptococcus equi* subsp. *zooepidemicus* (2.5)
13	*Streptococcus equi* subsp. *zooepidemicus* (99.9%)	*Streptococcus equi* subsp. *zooepidemicus* (2.4)
14	*Streptococcus equi* subsp. *zooepidemicus* (99.9%)	*Streptococcus equi* subsp. *zooepidemicus* (2.6)
15	*Streptococcus equi* subsp. *zooepidemicus* (99.9%)	*Streptococcus equi* subsp. *zooepidemicus* (2.3)
16	*Streptococcus equi* subsp. *zooepidemicus* (99.9%)	*Streptococcus equi* subsp. *zooepidemicus* (2.5)
17	*Streptococcus equi* subsp. *zooepidemicus* (99.9%)	*Streptococcus equi* subsp. *zooepidemicus* (2.6)
18	*Streptococcus equi* subsp. *zooepidemicus* (99.9%)	*Streptococcus equi* subsp. *zooepidemicus* (2.5)
19	*Streptococcus equi* subsp. *zooepidemicus* (99.9%)	*Streptococcus equi* subsp. *zooepidemicus* (2.3)
20	*Streptococcus equi* subsp. *zooepidemicus* (99.9%)	*Streptococcus equi* subsp. *zooepidemicus* (2.6)
21	*Streptococcus equi* subsp. *zooepidemicus* (99.9%)	*Streptococcus equi* subsp. *zooepidemicus* (2.6)
22	*Streptococcus equi* subsp. *zooepidemicus* (99.9%)	*Streptococcus equi* subsp. *zooepidemicus* (2.5)
23	*Streptococcus equi* subsp. *zooepidemicus* (99.9%)	*Streptococcus equi* subsp. *zooepidemicus* (2.6)

* Percent probabilities are reported in brackets. ** Scores are reported in brackets.

**Table 2:** Antibiotics and antibiotic classes tested for equine *S. zooepidemicus* isolates.

Antibiotics	ug	Antibiotics by Class	Reference for Breakpoints
Amoxicillin-clavulanate (AMC)	20/10	Penicillins (alone or combined)	[51]
Ampicillin (AMP)	30		[51]
Penicillin (P)	10 IU		[51]
Ceftiofur (EFT)	30	Cephalosporins	[51]
Ceftriaxone (CRO)	30		[52]
Amikacin (AK)	30	Aminoglycosides	[51]
Gentamicin (CN)	10		[51]
Kanamycin (K)	30		[51]
Streptomycin (S)	10		[51]
Imipenem (IMI)	10	Carbapenems	[51]
Meropenem (MRP)	10		[52]
Enrofloxacin (ENR)	5	Fluoroquinolones	[51]
Sulfamethoxazole-trimethoprim (SXT)	1.25/23.75	Sulfonamides	[52]
Tetracycline (TE)	30	Tetracyclines	[51]

## Data Availability

Not applicable.

## Supplementary Materials

The following supporting information can be downloaded at: https://www.mdpi.com/article/10.3390/antibiotics11010025/s1, Supplementary Table S1: *S. zooepidemicus* isolates growth inhibition zone values (mm) to tested antibiotics.
