# Supplementary material for: Occurrence and Antimicrobial Susceptibility Profiles of Streptococcus equi subsp. zooepidemicus Strains Isolated from Mares with Fertility Problems
Source: Antibiotics (Basel). 2021 Dec 27;11(1):25. doi: 10.3390/antibiotics11010025 (PMC8773361; doi:10.3390/antibiotics11010025)
Supplement: Supplementary file 1 [file antibiotics-11-00025-s001.zip › antibiotics-1495840-supplementary.pdf]

Article

Occurrence and Antimicrobial Susceptibility Profiles of *Streptococcus equi* subsp. *zooepidemicus* Strains Isolated from Mares with Fertility Problems

Francesca Paola Nocera [1,*], Elena D'Eletto [1], Monica Ambrosio [1], Filomena Fiorito [1], Ugo Pagnini [1] and Luisa De Martino [1,2]

[1] Department of Veterinary Medicine and Animal Production, University of Naples "Federico II", Via F. Delpino 1, 80137 Naples, Italy; elena.deletto93@gmail.com (E.D.); monica.ambrosio@unina.it (M.A.); filomena.fiorito@unina.it (F.F.); upagnini@unina.it (U.P.); ldemarti@unina.it (L.D.M.)

[2] Task Force on Microbiome Studies, University of Naples "Federico II", 80137 Naples, Italy

* Correspondence: francescapaola.nocera@unina.it; Tel.: +39-081-253-6182

Table S1. *S. zooepidemicus* isolates growth inhibition zone values (mm) to tested antibiotics.

ID	AMC	AMP	P	EFT	CRO	AK	CN	K	S	IMI	MRP	ENR	SXT	TE
**1**	17	22	17	31	30	---	---	NA*	---	15	24	23	NA	19
**2**	14	30	17	27	32	---	----	NA*	---	12	25	19	16	----
**3**	16	23	12	25	28	----	17	14	NA*	11	22	17	24	NA*
**4**	17	30	16	24	30	----	18	----	NA*	10	13	21	---	----
**5**	32	30	12	20	18	----	16	----	----	16	20	16	----	---
**6**	15	23	12	24	26	----	18	----	----	14	23	18	----	---
**7**	12	30	11	32	29	10	20	12	----	20	24	17	26	29
**8**	15	17	13	20	20	----	14	---	----	----	16	12	19	13
**9**	12	22	24	23	23	---	14	---	---	14	19	16	17	24
**10**	17	21	25	23	24	---	15	---	---	16	23	12	---	13
**11**	16	24	24	24	27	---	15	---	---	16	22	17	---	14
**12**	30	30	12	---	14	11	---	----	----	---	11	14	16	14
**13**	16	20	25	22	26	---	17	---	---	14	18	20	---	11
**14**	15	23	26	22	22	----	---	----	---	18	23	14	---	12
**15**	17	32	26	29	29	---	9	----	----	20	28	16	NA	23
**16**	29	21	28	26	32	---	19	10	---	20	12	17	16	---
**17**	14	23	23	10	15	---	---	----	----	----	10	17	---	11
**18**	17	20	30	27	28	17	20	18	18	22	22	16	17	NA*
**19**	12	20	23	23	19	---	11	NA*	---	14	19	15	--	---
**20**	16	22	23	32	22	---	---	---	----	26	20	16	---	27
**21**	27	22	28	25	27	---	15	---	NA*	20	NA*	NA*	23	--
**22**	15	23	24	26	24	---	---	----	---	NA*	26	14	---	---
**23**	17	23	19	32	32	---	---	---	---	18	22	---	19	20

* NA: not applicable. Antibiotics: AMC: amoxicillin-clavulanate; AMP: ampicillin; AK: amikacin; EFT: ceftiofur; CRO: ceftriaxone; ENR: enrofloxacin; CN: gentamicin; K: kanamycin; IMI: imipenem; MRP: meropenem; P: penicillin; S: streptomycin; SXT: sulfamethoxazole-trimethoprim; TE: tetracycline.
